# Exploring the efficacy of new potential bio-insecticides produced by *Penicillium oxalicum* against *Culex pipiens* larvae

**DOI:** 10.1186/s12866-026-04783-5

**Published:** 2026-02-25

**Authors:** Hayam A. E. Sayed, Enas H. S. Ghallab, Ahmed M. Elissawy, Peter F. Farag, Nevin A. Ibrahim

**Affiliations:** 1 Department of Microbiology, Faculty of Science, Ain Shams University, Cairo, 11566 Egypt; 2 Department of Entomology, Faculty of Science, Ain Shams University, Cairo, 11566 Egypt; 3 Department of Pharmacognosy, Faculty of Pharmacy, Ain Shams University, Cairo, 11566 Egypt

**Keywords:** *Culex pipiens*, Larvicidal activity, Statistical optimization, Molecular docking, NMR spectroscopy, Mangrove-associated fungi.

## Abstract

**Background:**

Synthetic insecticides are major contributors to environmental pollution and the emergence of resistance. Fungal-derived metabolites are known to be environmentally friendly, selective alternatives. Thus, in the context of a green environment, this study aimed to evaluate the larvicidal activity of a mangrove-associated endophytic *Penicillium oxalicum* (OQ231606.1) against *Culex pipiens* using a larval mortality bioassay and molecular docking.

**Results:**

The culture filtrate extract of the examined *P. oxalicum* OQ231606.1 strain caused up to 80% mortality (optimized to 99.98%). Metabolite production conditions were optimized using a full factorial design followed by a central composite design. Optimum conditions were pH 6, 1.2 × 10^6^ spores/mL inoculum, 28 °C, for 20 days. Physcion and deoxybrevianamide E, two bioactive compounds with newly reported biolarvicidal activity, were isolated from the fungal extract and structurally elucidated via NMR spectroscopy. *C. pipiens* third-instar larvae treated with various concentrations of these compounds showed increased mortality with increasing concentrations. Notably, deoxybrevianamide E was more potent, with an LC₅₀ value less than 1 µg/mL at 48 h (LC_50_ = 0.84 µg/mL vs. Physcion’s = 12.64 µg/mL). Docking analysis revealed potential insect receptors interacting with candidate compounds, showing deoxybrevianamide E and okaramine B (control) share the same active site, targeting glutamate-gated chloride channel (GluCl) with binding affinities of − 9.5 Kcal/mol and − 9.1 Kcal/mol, respectively. Whereas physcion and emodin (control) target acetylcholinesterase (AChE), with binding affinities of − 8.5 Kcal/mol and − 8.9 Kcal/mol, respectively.

**Conclusion:**

This study highlights *P. oxalicum* OQ231606.1 as a sustainable reservoir for bioinsecticide development, identifying physcion and deoxybrevanamide E as bioactive compounds with newly reported biolarvicidal activity that represent promising candidates for green vector control strategies.

**Supplementary Information:**

The online version contains supplementary material available at 10.1186/s12866-026-04783-5.

## Introduction

Mosquitoes are small, flying insects known for their ability to transmit diseases such as malaria, dengue, and the Zika virus, making them one of the most significant vectors of human pathogens worldwide. The northern house mosquito *Culex pipiens* Linnaeus (Diptera: Culicidae) is a widely distributed vector capable of transmitting pathogens to both humans and animals, notably medically significant viruses such as West Nile virus and Japanese encephalitis virus [1]. Controlling mosquito populations typically involves preventing their breeding through the use of various insecticides that target different life stages [2].

The use of conventional chemical pesticides is increasingly restricted due to their negative environmental impact and the rapid emergence of pest resistance, leading to an estimated annual decline of approximately 2% in their global use. In contrast, the application of biopesticides, including bioinsecticides, has expanded markedly, with reported annual growth rates of around 10% [3]. Although alternative non-insecticidal approaches, such as Attractive Toxic Sugar Baits (ATSB), have demonstrated high efficacy and minimal impact on non-target organisms [4, 5], the continued discovery of novel fungal-derived bioactive compounds remains essential. The development of such biopesticides is critical for broadening the Integrated Pest Management (IPM) toolbox and for sustaining effective control of mosquito populations in the face of increasing insecticide resistance [6].

All categories of biopesticides, including microbial biopesticides, biochemical biopesticides, and plant-incorporated protectants (PIPs), together account for about 5% of the global pesticide market, with microbial biopesticides being the top priority [7]. Biopesticides are eco-friendly, biodegradable, and can overcome resistance caused by synthetic pesticides, in addition to their specific action against only their target organisms [8]. Bioinsecticides have been utilized for the control of *Culex* mosquitoes for several decades [9–11]. Mosquito larvicides are derived from various microorganisms, including bacteria such as *Bacillus thuringiensis* (Bt) and *Lysinibacillus sphaericus*, as well as fungi like *Beauveria bassiana* and *Metarhizium anisopliae* [12].

Mangrove ecosystems have received significant attention due to their distinctive environmental conditions, including high salinity, muddy or sandy soils, low pH, and partial anoxia, which are periodically flooded by tidal waters [13]. These ecosystems are hotspots for diverse microorganisms, and the uniquely harsh environment of mangroves encourages the production of new and potent bioactive metabolites through their associated microbiota. In particular, mangrove-derived fungi, especially endophytic species, possess distinctive metabolic pathways that allow them to generate a diverse array of secondary metabolites [14–18]. These metabolites often exhibit unique structures and significant biological activities, including strong insecticidal properties. As a result, they have promising applications in various fields, such as medicine, agriculture, bioremediation, and pest biocontrol [19–24]. Consequently, the environmentally friendly properties of these distinctive biodegradable fungal metabolites make mangrove-associated fungi promising candidates for developing novel insect biocontrol agents, which enhance integrated vector management.

As a revolutionary method in pesticide discovery, in-silico molecular docking provides intricate insights into the interactions between natural compounds (ligands) and potential biological targets (mostly proteins). The relevance of molecular docking in insect pest management is investigated, showing its efficiency in identifying novel targets quickly and without cost [25–27]. With an ongoing increase in insecticide resistance due to the one-target mechanism of action, inverse molecular docking represents a unique approach utilized in the screening of potential multiple target proteins for a given natural product [28–30]. Multitarget insecticides are more likely to achieve comprehensive regulation of insects with a low risk of resistance, in which ligands selected for a particular target protein may unintentionally inhibit other proteins within that pathway or a ligand may bind multiple proteins from different pathways [21–33].

This study aimed to screen crude extracts of locally isolated mangrove-derived endophytic fungal isolates for their potential larvicidal effects against *C. pipiens* larvae. It focused on optimizing both physical and cultural growth conditions to maximize the production of bio-larvicidal metabolites by the most potent isolate. Two different statistical approaches were used: a 2^k^ full factorial design (FFD)and a central composite design (CCD). Furthermore, the study included the isolation and purification of bioactive compounds, elucidation of their chemical structures, and verification of their efficacy using a larval mortality bioassay. In silico molecular docking of the purified compounds and selected target insect protein was also performed to better understand their potential mechanism of larvicidal action.

## Materials and methods

### Screening fungal isolates for their larvicidal potential

#### Test fungi and culture maintenance

The six endophytic fungal isolates studied, namely *Chaetomium globosum*,* Stachybotrys sp.*,* Aureobasidium sp.*,* Cladosporium cladosporioides*,* Aspergillus terreus*, and *Penicillium oxalicum* OQ231606.1, were kindly provided by Dr. Mohamed A. Abuzeid, Professor of Microbiology at Ain Shams University, and his colleagues. These endophytic fungi, isolated from the grey mangrove *Avicennia marina*, were part of a thesis by Heba S. Salem under Dr. Abuzeid’s supervision [34]. The strain *P. oxalicum* (OQ231606.1) was recognized as the most bioactive isolate and selected for study optimization. Its taxonomic identity was molecularly confirmed by Salem [34], using ITS1-5.8 S-ITS2 sequencing, with BLAST and phylogenetic analyses showing > 99% similarity to established species. The sequence data is deposited in the NCBI GenBank database under accession number OQ231606.1, and are publicly available at: “https://www.ncbi.nlm.nih.gov/nuccore/OQ231606.1?report=genbank”. Isolates were cultured on Potato Dextrose Agar (PDA; Difco, Sparks, MD, USA) and incubated at 25 °C for 5 to 7 days before being stored at -4 °C till further use.

#### Fermentation and preparation of fungal crude extracts

The six fungal isolates were cultivated in 250 mL Erlenmeyer flasks containing 50 mL of modified Malt Extract Broth medium (MEB; HiMedia, Mumbai, India) composed of malt extract 6 g/L, maltose 1.8 g/L, dextrose 6 g/L, and yeast extract 1.2 g/L), and incubated at 25 °C for 7 days in a shaking incubator (150 RPM). After the incubation period, each isolate was filtered separately under aseptic conditions, and the fungal biomass was removed by passing the broth culture through Whatman filter paper. No.1 (Sigma, St. Louis, MO, USA). Each fungal growth-free supernatant was extracted using an equal volume (1:1 v/v) of ethyl acetate (EtOAc; Sigma, St. Louis, MO, USA) in three separate extractions. The combined organic extract was then dried using a rotary evaporator at 40 °C. The prepared crude extracts were dissolved in 0.1% (v/v) dimethyl sulfoxide (DMSO; Sigma, St. Louis, MO, USA) and stored at -20 °C for future evaluation of their bioactivity [35, 36]. All experiments were performed in three replicates, and the mean values and standard deviations were determined.

#### Assessing bioactivity through larval mortality bioassay

##### Mosquito collection and preparation

Third-instar larvae of *Culex pipiens* Linnaeus (L.) (Diptera: Culicidae) were collected from their known natural breeding sites in Abou Rawash, Giza, Egypt, using a standard dipper. The larvae were carefully transported to the lab in containers filled with water from the collection site to minimize stress. In the lab, the larvae were carefully identified morphologically and sorted under a stereomicroscope; only those in the third instar stage were selected for further testing [37]. Larvae were maintained in trays with dechlorinated water and fed on a diet of ground fish food under rearing conditions of 28^ᵒ^C, 80% RH and daylight period of 14 h [38]. For our experiments, we used only third-instar larvae that were obtained either directly from the field (when already at that stage upon collection) or from laboratory-reared cohorts that originated from 

##### Bioassay set up

Preliminary screening for larvicidal activity was conducted by applying the prepared fungal crude extracts to the third-instar larvae, based on the standard World Health Organization procedure [39] with slight modifications. Groups of 25 larvae were placed in 100 mL of dechlorinated tap water in small plastic cups. Each group was treated with a fungal crude extract, and each treatment was replicated three times to ensure consistency. The larvae were immersed in the solution for 48 h, during which no food was provided. We monitored larval mortality at specific intervals, namely after 24 and 48 h. Larvae were considered dead if they no longer moved or failed to respond to gentle prodding, and their numbers were recorded accordingly. A control group of third-instar larvae exposed to 0.1% (v/v) DMSO in water was used as a negative control. Mortality in this control group was monitored in the same way as the treated groups. Experiments were conducted three times at 28^ᵒ^C, 80% RH and daylight period of 14 h, and the mean values and standard deviations were determined.

### Optimizing production of bioactive compounds

#### Analyzed factors and experimental design

Four independent factors, including one physical factor (temperature), one chemical factor (pH), along with two cultural factors (incubation period and inoculum size), were used to optimize fungal active compound(s) production by the selected most potent fungus. The response, larval mortality percentage, was recorded after each run and analyzed through experimental design. The process had two stages: first, exploring main effects and interactions using a 2^4^ full factorial design. In this approach, all possible combinations of the factor levels (high and low) were tested. Factors, codes, levels, and units are in Table 1; the design summary is in Table 2. Next, a response surface methodology (RSM), specifically the central composite design (CCD), was used to develop an optimization model. CCD improves upon the factorial design by incorporating center points for error detection and axial points for quadratic effects. This allows us to estimate model curvature. The tested factor levels, along with codes and units, are shown in Table 3, and the CCD point distribution is summarized in Table 4.

**Table 1:** The tested factors in FFD* and their codes, levels, and units

Variable	Code	Low level (-1)	High level (+1)	Unite
pH	A	5	8	-
Inoculum size	B	4	20	%
Temperature	C	25	30	ºC
Incubation period	D	15	30	Days

* Full factorial design

**Table 2:** 2^4^ FFD* Summary

Factors:	4	Base Design:	4, 16
Runs:	32	Replicates:	2
Blocks:	1	Center pts (total):	0

* Full factorial design

**Table 3:** The tested factors in CCD** with their codes, levels and units

Variable	Code	-2	-1	0	+1	+2	Unite
pH	A	3.5	5	6.5	8	9.5	-
Inoculum size (I.S)	B	0	4	12	20	28	%
Temperature (Tem)	C	22.5	25	27.5	30	32.5	ºC
Incubation period (I.P)	D	7.5	15	22.5	30	37.5	Days

**Central composite design

**Table 4:** The CCD** summary and point distribution

Design Summary				Point Types	
Factors:	4	Replicates:	3	Cube points:	48
Base runs:	30	Total runs:	90	Center points in cube:	12
Base blocks:	2	Total blocks:	2	Axial points:	24
				Center points in axial:	6

**Central composite design

### Fermentation procedure and inoculum preparation

The codes, levels, and units of the selected optimization factors, as well as the experimental design, are summarized in Tables 1, 2, 3 and 4; otherwise, the fermentation conditions were identical to those described above in the screening section regarding the fermentation methodology. The pH of the Malt extract broth (MEB) media was adjusted to the desired levels using 1% hydrochloric acid (HCl) and 1 N sodium hydroxide (NaOH). The required pH values were monitored using a pH meter (Walk LAB Microprocessor pH meter HP9000). For inoculum preparation, each tested fungus was grown on Malt Extract Agar (MEA; HiMedia, Mumbai, India) for 7 days. After the incubation period, a spore suspension with an initial concentration of approximately 1 × 10^7^ spores/mL was prepared. This spore suspension was used as a stock to prepare the required inoculum concentrations through serial volume/volume dilutions to achieve the desired inoculum sizes, according to the method described by El-Shazly et al. [40]. Finally, after each fermentation run, the larvicidal activity of the obtained crude extracts was assayed, similar to that mentioned above in the “Larval Mortality Bioassay” section.

### Models’ generation and statistical analysis

Both the 2^4^ full factorial design and the Central Composite Design (CCD) models were created using the Minitab^®^ 21 software package [41]. The results obtained were analyzed using ANOVA to assess the significance of each model and its terms. The *P*-values were used to determine which terms (main effects, interaction effects, and quadratic terms) were statistically significant. The goodness of fit of the model was reported from the R-squared (R²) value of each model. Finally, optimization of the active compound production was achieved using Minitab’s optimization tools.

### Purification and structural elucidation of bioactive compound (s)

#### General experimental procedures

NMR spectra were recorded on a Bruker^®^ AVANCE III HD, 400 MHz spectrometer (Zürich, Switzerland). Final purification steps were done using Shimadzu HPLC semi-preparative series combined with UV-PDA (photodiode array): SPD-M20A (column: 250 × 10 mm), Kromasil 100-10-C18 reversed-phase (RP-C-18). VLC fractionation was conducted using a Vacuubrand^®^ vacuum pump (Germany), and normal phase column chromatography was performed using Silica Gel 60 (0.04–0.063, Merck, Germany).

#### Isolation of pure compounds

Following the completion of the fermentation process, the fermentation supernatant was extracted using 600 mL of EtOAc three times and then filtered. The obtained crude extract (800 mg) was partitioned between n-hexane and 90% aqueous methanol, yielding approximately 600 mg of the 90% aqueous methanol extract. Subsequently, the 90% methanolic fraction was applied to a vacuum liquid chromatography (VLC) assembly packed with normal stationary phase silica gel 60, and elution was performed using a gradient mobile phase n-hexane: ethyl acetate (100:0 to 0:100), followed by dichloromethane: methanol (100:0 to 0:100) to give 8 fractions. The collected fractions were subsequently investigated using TLC with various mobile phases. Finally, after pooling similar fractions, 4 fractions were obtained. Fractions L2 and L3 were selected for further purification procedures. Fraction L2, eluted with EtOAc /n-hexane (30:70%), was finally purified on preparative HPLC eluted with a H_2_O: Acetonitrile mixture 75% to yield compound 1 (6 mg). Moreover, the second major subfraction of L3 was applied to a semi-preparative HPLC C-18 column for additional purification, yielding 2 (5 mg) and 3 (3 mg).

### Larvicidal efficacy of the purified compound (s)

The purified compounds obtained were tested for their larvicidal activity using the aforementioned larval mortality bioassay. Each group of larvae was treated with different concentrations of the purified compound (0.315, 0.625, 1.25, 2.5, 5 µg/ml for deoxybrevianamide E, and 3.25, 6.2, 13, 26, 52 µg/ml for physcion), which was dissolved in 0.1% (v/v) DMSO in water. To ensure consistency, each treatment was replicated three times, and a negative control group (0.1% DMSO in water) was included. Larval mortality was recorded after 24 and 48 h. Mortality data were recorded in a probit regression line. LC_50_ and slope values were calculated.

#### Statistical analysis

Pairwise comparisons were performed between 24 h and 48 h mortality rates at each concentration to assess time-dependent toxicity. Comparisons were conducted using the Chi-square (χ²) test of independence. Fisher’s exact test was applied when expected frequencies were below five. Statistical analyses were performed using IBM SPSS Statistics software, version 28.0 (IBM Corp., Armonk, NY, USA) [42].

### Molecular docking analysis

The 3D structures of the bioactive compounds (Physcion and Deoxybrevianamide E) were retrieved from the PubChem in sdf format. At the same time, UCSF ChimeraX v1.6.1 [43] was used to extract the 3D structures in mol2 format. From the protein data bank, insecticide-resistant acetylcholinesterase (PDB ID: 6ARY), nicotinic acetylcholine receptor (PDB ID: 2BG9), glutamate-gated chloride channel (PDB ID: 3RHW), odorant-binding Protein (PDB ID: 3OGN), and chitin deacetylase (PDB ID: 5ZNT) were downloaded. UCSF ChimeraX is used to remove ligands, ions, and water molecules from the receptors. For primary virtual screening, ReverseDock web server [44] was utilized to perform inverse molecular docking of each ligand (mol2) and multiple target proteins. Following the selection of the best protein-ligand complexes, a molecular docking analysis was performed using CB-Dock2 [45] to investigate the interaction bonds between the ligands and receptors. The dock complexes were visualized using ChimeraX/ISOLDE software.

### Prediction of compounds’ toxicity

Toxicity properties of deoxybrevianamide E and physcion were evaluated using ProTox 3.0 [46]. The platform is organized into four categories: organ toxicity (5 models), toxicity endpoints (8 models), toxicity pathways (12 models), and toxicity targets (15 models). The acute toxicity prediction for oral administration within 24 h was reported as predicted lethal dose-50 (LD_50_) (mg kg^− 1^ body weight). LD_50_ was utilized for further study of toxicity classes, which are determined according to the globally harmonized system (GHS) of categorization and labeling of substances (LD_50_ values are reported in mg kg^− 1^). class I: fatal if consumed (LD_50_ ≤ 5); class II: fatal if consumed (5 < LD_50_ ≤ 50); class III: toxic if swallowed (50 < LD_50_ ≤ 300); class IV: harmful if swallowed (300 < LD_50_ ≤ 2000); class V: may be harmful if swallowed (practically non-toxic; 2000 < LD_50_ ≤ 5000); class VI: non-toxic (LD_50_ > 5000) [47].

## Results

### Primary screening of larvicidal activity and general impact on *Culex pipiens* larvae

**Fig. 1:**
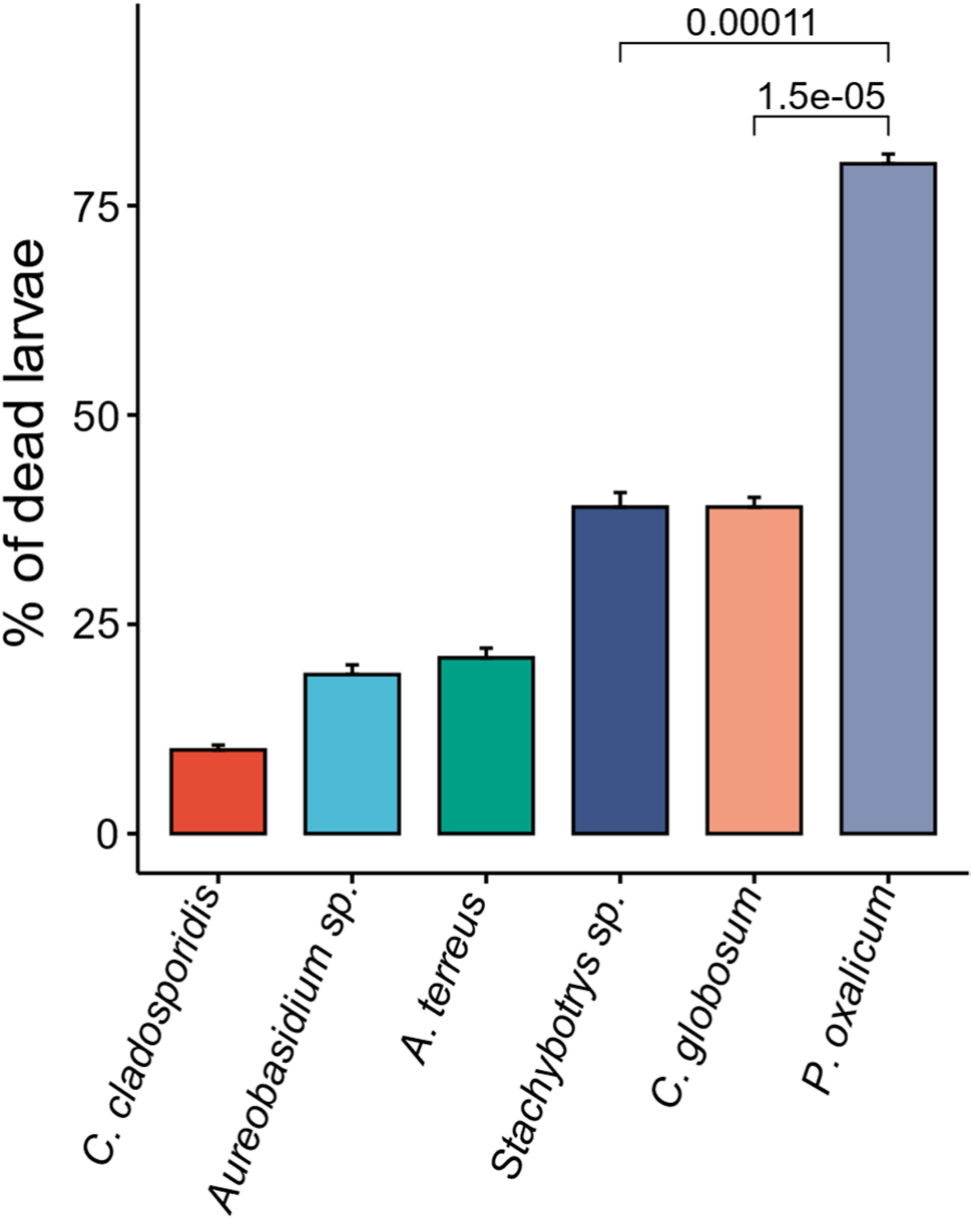
Larvicidal activity of the screened endophytic fungi, showing a highly significant difference between the test isolates (*P*-value < 0.001). *P*-value was calculated using the Wilcoxon test

**Fig. 2:**
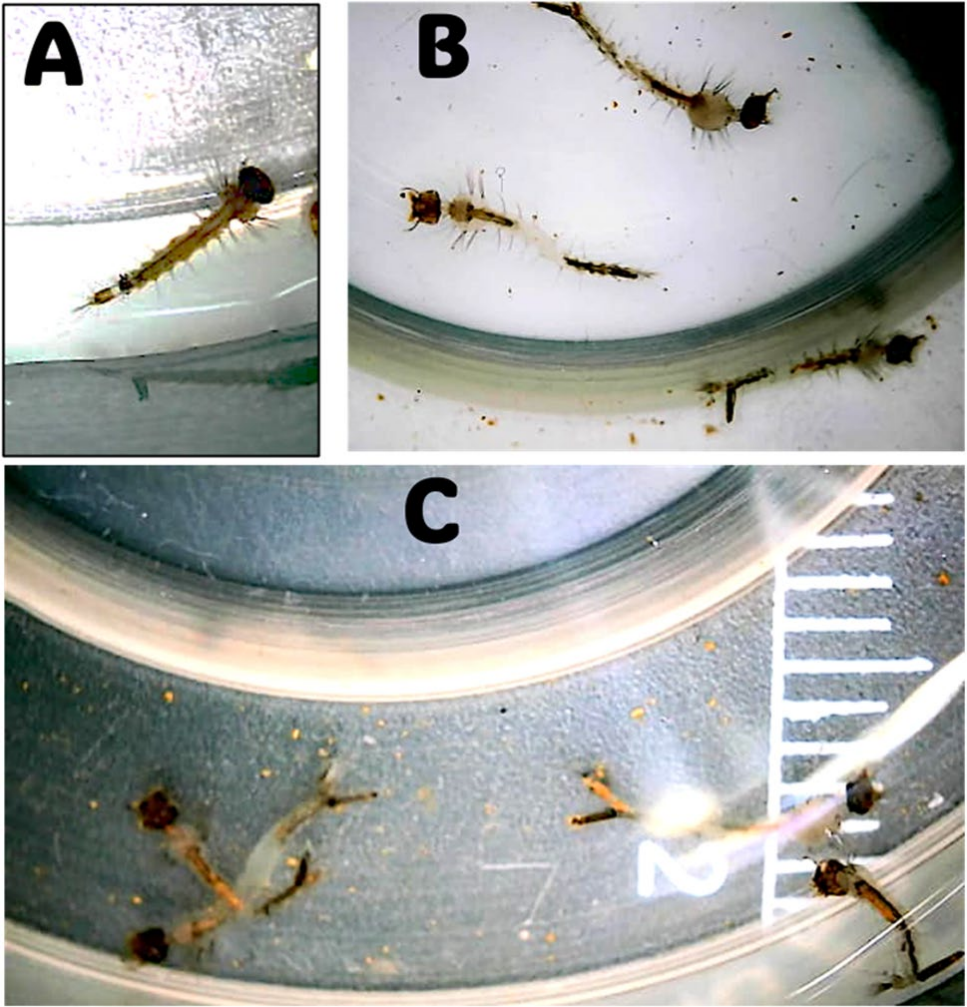
Morphological alterations in *Culex pipiens* larvae exposed to *Penicillium oxalicum* OQ231606.1 extract. **A** (Control): Untreated fourth-instar larva showing normal morphology, including an intact cuticle, uniform body segmentation, No external deformities or internal particulate matter is observed; **B** (showing elongated larva): Larva exposed to the fungal extract exhibiting body elongation, loss of cuticle rigidity, and rupture of the alimentary canal; **C** (showing fungal extract particles): High transparency in a treated larva showing accumulation of fungal extract particles (yellow particles) within the midgut lumen and hemocoel

The primary screening of six tested endophytic fungi for their larvicidal activity revealed varying levels of efficacy in killing *C. pipiens* larvae, with mortality rates ranging from 11% to 80%. The crude extract of *Penicillium oxalicum* OQ231606.1 was the most active, showing a significantly high mortality rate of up to 80%, as illustrated in Fig. 1. Consequently, this fungal strain was selected for further optimizing its growth conditions to enhance the production of larvicidal compounds. Notably, when exposed to the *P. oxalicum* OQ231606.1 extract, the *C. pipiens* larvae exhibited significant morphological changes. The tested larvae displayed physical deformities, including severe elongation and increased transparency, as well as visible accumulation of fungal extract particles within their bodies (Fig. 2).

### Optimization of bioactive compound production by *P. oxalicum*

#### 2^4^ full factorial design

The most potent fungal isolate, *P. oxalicum* OQ231606.1, was selected for further optimizing its fermentation conditions to maximize the production of larvicidal metabolites. To investigate the impact of individual factors and their interactions on the production of active compounds, we initiated the optimization process using a 2^4^ FFD. The design matrix, which includes the studied variables in uncoded values, as well as the observed and predicted percentages of larval mortality following treatment with crude extracts of culture filtrate, as shown in Table 5. The highest response was recorded during run 5 and its replicate. In this case, all tested factors were at low levels, except for the incubation temperature, which was set at a high level. Referring to the coded coefficients in the Supplementary Table S1, we found that two out of the four investigated factors had a significant negative effect on production when moving from low to high levels. In contrast, only the temperature significantly increased when transitioning from low to high levels. The change in inoculum size resulted in a slight, non-significant increase in response. In the designed model, we obtained a high coefficient of determination, R2 (97.76%), which indicates that the investigated parameters explain 97.76% of the variations in the larvicidal compound(s) production levels, with only 2.24% unexplained. The results of the model were fitted into the following polynomial regression equation:

$$\begin{aligned}Death\_\%=&-1236\;+142.0\;pH\;+66.0\;I.S\;+50.12\;Tem\;\\&+46.76\;I.P\;-9.11\;pH\ast I.S\;-5.683\;pH\ast Tem\;\\&-5.711\;pH\ast I.P\;-2.529\;I.S\ast Tem\;-2.703\;I.S\ast I.P\;\\&-1.861\;Tem\ast I.P\;+0.3458\;pH\ast I.S\ast Tem\;\\&+0.3889\;pH\ast I.S\ast I.P\;+0.2244\;pH\ast Tem\ast I.P\;\\&+0.1018\;I.S\ast Tem\ast I.P\;-0.01444\;pH\ast I.S\ast Tem\ast I.P\end{aligned}$$

**Table 5:** 2^4^ FFD* matrix showing the observed and expected death percent of mosquito larvae upon 24-hour treatment with fungal extract

Run	Variables				The Response (% of death)	
	pH	I.S	Temp	I.P	Observed	Expected
1	5	4	25	15	12	14
2	8	4	25	15	8	8
3	5	20	25	15	17	17
4	8	20	25	15	8	9
5	5	4	30	15	58	60
6	8	4	30	15	26	27
7	5	20	30	15	32	34
8	8	20	30	15	30	30
9	5	4	25	30	9	9
10	8	4	25	30	4	4
11	5	20	25	30	6	7
12	8	20	25	30	27	19
13	5	4	30	30	8	8
14	8	4	30	30	14	13
15	5	20	30	30	13	13
16	8	20	30	30	16	16
17	5	4	25	15	15	14
18	8	4	25	15	8	8
29	5	20	25	15	16	17
20	8	20	25	15	9	9
21	5	4	30	15	61	60
22	8	4	30	15	28	27
23	5	20	30	15	36	34
24	8	20	30	15	30	30
25	5	4	25	30	8	9
26	8	4	25	30	3	4
27	5	20	25	30	8	7
28	8	20	25	30	12	19
29	5	4	30	30	8	8
30	8	4	30	30	13	13
31	5	20	30	30	14	13
32	8	20	30	30	15	16

* Full factorial design

**Fig. 3:**
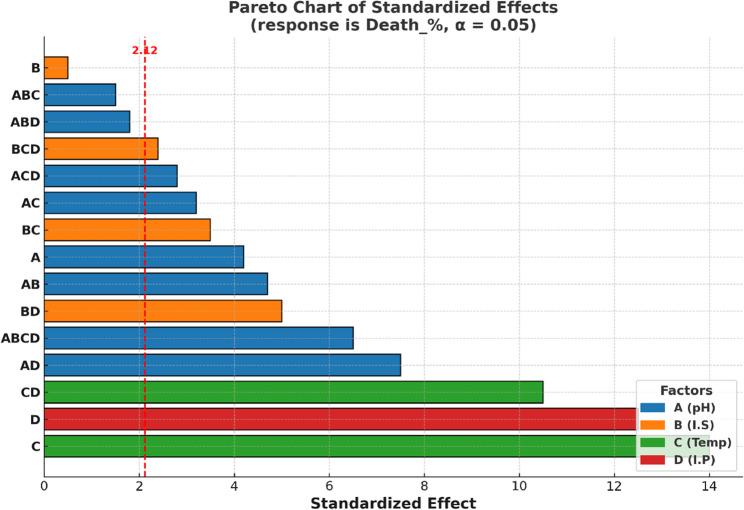
Pareto chart of standardized effects of different factors and their interactions in the 2^4^ FFD

**Fig. 4:**
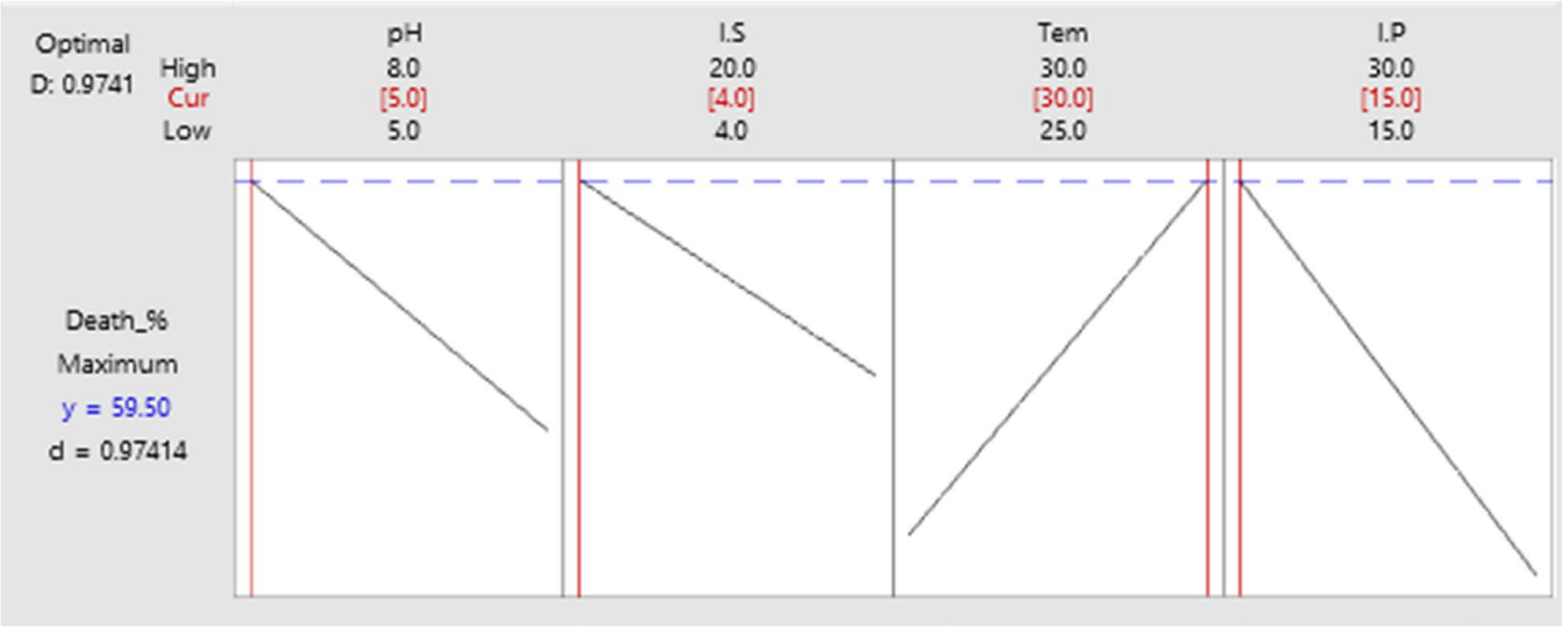
The predicted optimum conditions from FFD model for the highest production level

The model is highly significant (*P* < 0.05, F = 188.31), indicating it explains variability in response (Supplementary Table S2). Production of bioactive compound(s) is influenced by factors and their interactions, up to four-way interactions. Three variables: pH (F = 17.88), temperature (F = 196.44), and inoculum size (F = 170.28) significantly affect production (*P* = 0.000), with inoculum size showing no main effect but significant interactions (F = 0.13, *P* = 0.722). Although inoculum size had no significant impact on the production level, it couldn’t be ignored in the model because when interacted with other factors, either two-way or three-way interactions, it showed a *P* < 0.05 as displayed in the Pareto chart (Fig. 3). Notably, pH*Tem*I.P and I.S*Tem*I.P are significant three-way interactions (*P* < 0.05). The four-way interaction pH*I.S*Tem*I.P is highly significant (F = 104.39, *P* = 0.000), indicating that this complex interaction is crucial for explaining the response. Using the response optimizer tool, optimal growth conditions were predicted at pH 5, 4% inoculum size, 15 days of incubation, and 30 °C, as shown in Fig. 4.

**Fig. 5:**
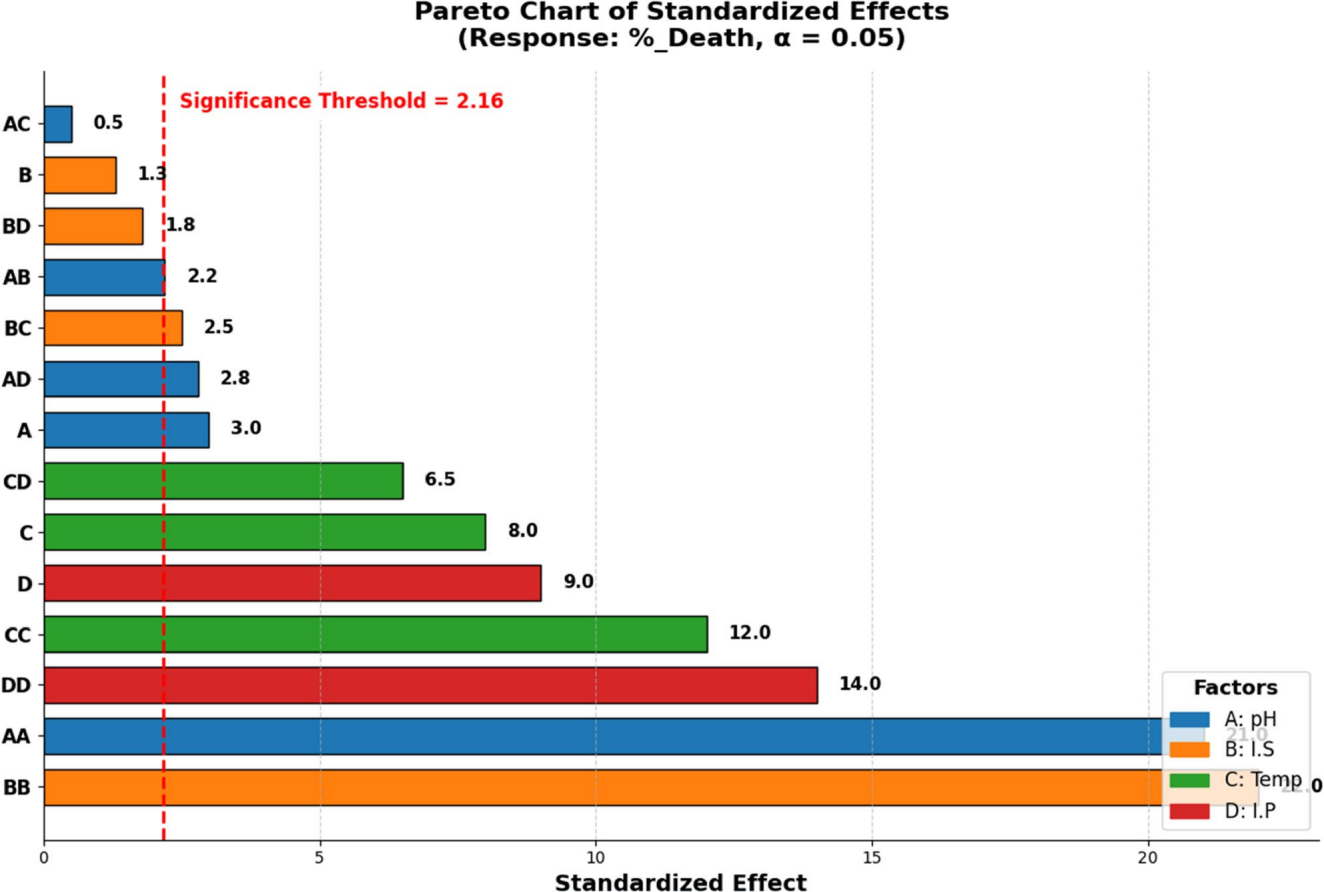
Pareto chart of standardized effects of different factors and their interactions in the CCD Multiple Response Prediction

#### Central composite design (CCD)

All investigated physical growth factors significantly affected larvicidal compound(s) production, either alone or in interactions, as tested by FFD. Thus, all of them were represented in the CCD for precise prediction. The design matrix (Table 6) shows variables in uncoded values, with observed and predicted larval mortality percentages after 24 h of treatment. Supplementary Table S3 reveals that inoculum size has a negligible effect alone or combined with other factors. pH and I.P. had a significant negative impact on production, while incubation temperature positively influences it. The model’s coefficient of determination (R^2^) of 98.71% suggests it explains most variation in production levels, with only 1.29% unexplained. ANOVA analysis confirms the CCD model’s high significance (*P* < 0.05, F = 62.02), indicating it efficiently explains variation in production levels (Supplementary Table S4). All linear (except I.S.) and quadratic terms are significant (*P* < 0.05), with Temperature and incubation period being most influential. The effect of the studied variables and their interactions is collectively demonstrated in the Pareto chart (Fig. 5). The model shows a non-significant lack of fit, implying it captures all the factors that affect the response. The findings of the model were fitted into the following regression equation:

$$\begin{aligned}\%\_Death=&-2437\;+135.6\;pH\;+10.77\;I.S\;+131.7\;Tem\;\\&+18.01\;I.P\;-11.204\;pH\ast pH\;-0.4017\;I.S\ast I.S\;\\&-2.193\;Tem\ast Tem\;-0.2659\;I.P\ast I.P\;+0.125\;pH\ast I.S\;\\&+0.033\;pH\ast Tem\;+0.256\;pH\ast I.P\;-0.0937\;I.S\ast Tem\;\\&+0.0229\;I.S\ast I.P\;-0.3333\;Tem\ast I.P\end{aligned}$$

**Table 6:** CCD** design matrix showing the observed and expected death percent of mosquito larvae upon 24-hour treatment with fungal extract

Run	Variables				Response (% of larval death)	
	pH	I.S	Temp.	I.P	Observed	Expected
1	8.0	4	25.0	15.0	8	7
2	5.0	20	25.0	15.0	17	18
3	5.0	4	30.0	15.0	61	51
4	8.0	20	30.0	15.0	30	31
5	5.0	4	25.0	30.0	8	8
6	8.0	20	25.0	30.0	1	12
7	8.0	4	30.0	30.0	9	9
8	5.0	20	30.0	30.0	4	6
9	6.5	12	27.5	22.5	100	97
10	6.5	12	27.5	22.5	98	97
11	5.0	4	25.0	15.0	15	18
12	8.0	20	25.0	15.0	8	5
13	8.0	4	30.0	15.0	28	33
14	5.0	20	30.0	15.0	32	35
15	8.0	4	25.0	30.0	2	1
16	5.0	20	25.0	30.0	8	5
17	5.0	4	30.0	30.0	3	8
18	8.0	20	30.0	30.0	6	5
19	6.5	12	27.5	22.5	100	93
20	6.5	12	27.5	22.5	94	93
21	3.5	12	27.5	22.5	6	7
22	9.5	12	27.5	22.5	0	-4
23	6.5	-4	27.5	22.5	0	2
24	6.5	28	27.5	22.5	2	-2
25	6.5	12	22.5	22.5	36	34
26	6.5	12	32.5	22.5	62	61
27	6.5	12	27.5	7.5	58	60
28	6.5	12	27.5	37.5	30	25
29	6.5	12	27.5	22.5	98	102
30	6.5	12	27.5	22.5	96	102

** Central composite design, The runs carried out in triplicate and the mean of responses used for the analysis

**Fig. 6:**
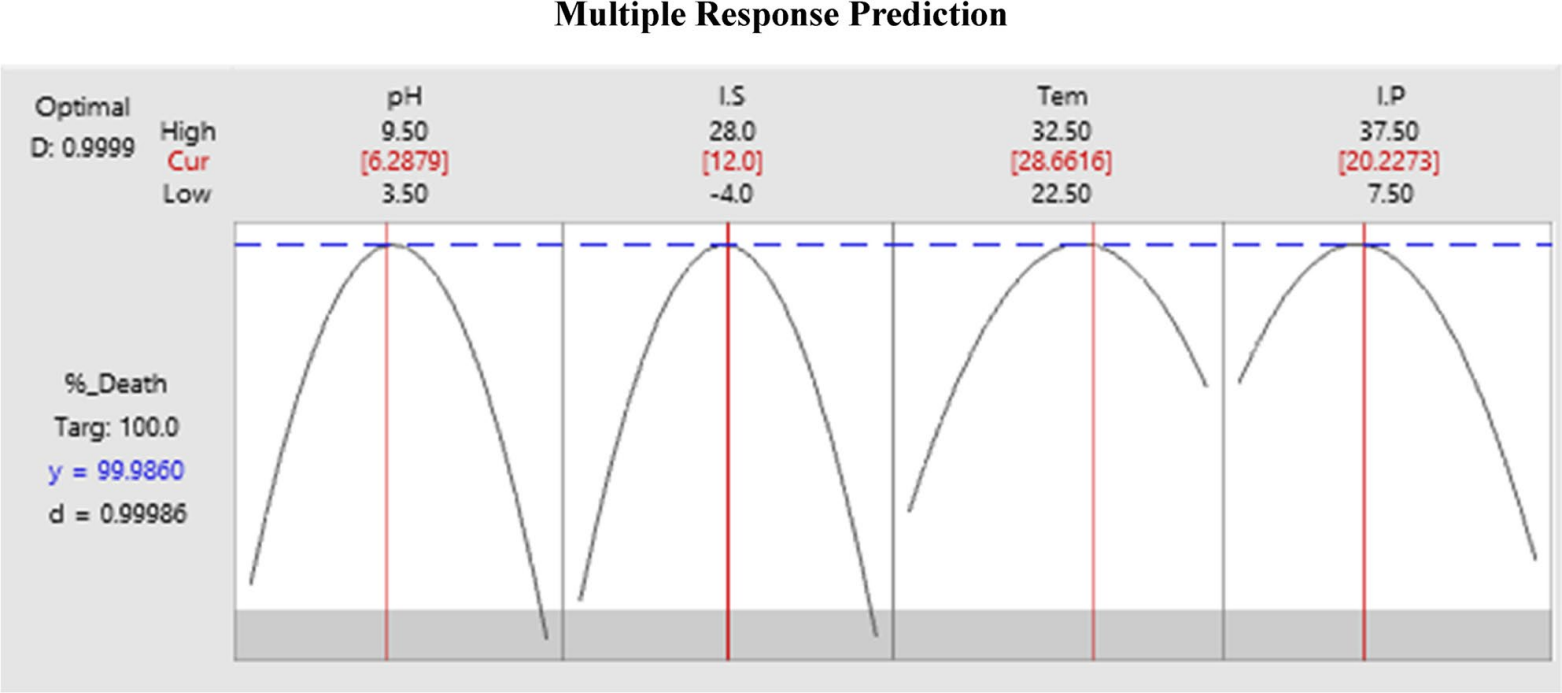
The predicted optimum conditions from the CCD model for the highest production level

##### Prediction of the optimum conditions

Predicting Optimal Conditions: The Response Optimizer tool was used to accurately predict the conditions required to produce the maximum level of larvicidal compound(s). It suggested the optimal level for each variable at a desirability of 0.99 with a 95% confidence interval. Predicted optimal conditions included: growth in a medium with a pH of approximately 6, using a 12% inoculum volume (~ 1 × 10^6^ spores/mL) and incubation at about 28 °C for 20 days as illustrated in Fig. 6.

### Purification and chemical characterization of bioactive compound (s)

Compound 1 was isolated as an orange solid. Interpretation of ^1^H and ^13^C NMR data (CDCl3; Supplementary Figures S1-S3 ) of compound 1, showed the presence of 2 chelating protons of hydroxyl groups (δH 12.33,s, H-5, δH 12.14,s, H-4,), 4 metacoupled aromatic protons of which H-1 (δH 7.65, broad signlet) showed weak COSY correlations ( Figure S3)(with H-3 (δH 7.11, brd s) as well as between H-6 at (, δH 6.71, d, 2.2) and H-8 (δH, 7.39, d, 2.2) additionally the proton NMR spectrum revealed the presence of two methyl peaks at δH 2.48 and the methoxy protons at δH 3.87. These features, together with the ¹³C NMR spectrum, align closely with previously reported data for physcion, (C_16_H_12_O_5_), an anthraquinone derivative, confirming the identity of compound 1.

Compound 2 was isolated as a yellowish solid. ¹H and ^13^ C NMR spectral analysis of compound 3 (CDCl_3_; Supplementary Figures S4 and S5) revealed the presence of 21 carbon signals classified into two methyls, 7 methines of which 5 are aromatic/olefenic carbons, 5 methylenes of which 4 are aliphatic and one is terminal olefinic methylene, and 7 quaternary carbons. These features correspond well with reported NMR data for deoxybrevianamide E (C_21_H_25_N_3_O_2_), a diketopiperazine alkaloid.

### Larvicidal activity and LC_50_of the purified compounds

Different concentrations of deoxybrevianamide E and physcion were applied to third-instar larvae of *C. pipiens*. Mortality data after 24 and 48 h were plotted in a probit regression line, and the LC_50_ and slope function were calculated. Mortality rates are shown in Supplementary Tables S5 and S6. The data in these tables indicate that mortality rates increased significantly with increasing concentrations of each compound. The lowest mortality rates (12.86% and 39.92%) were recorded at 0.315 µg/mL deoxybrevianamide E after 24 and 48 h, respectively. Whereas the lowest mortality rates (7.84% and 15.29%) were recorded at 3.25 µg/mL physcion after 24 and 48 h, respectively.

**Fig. 7:**
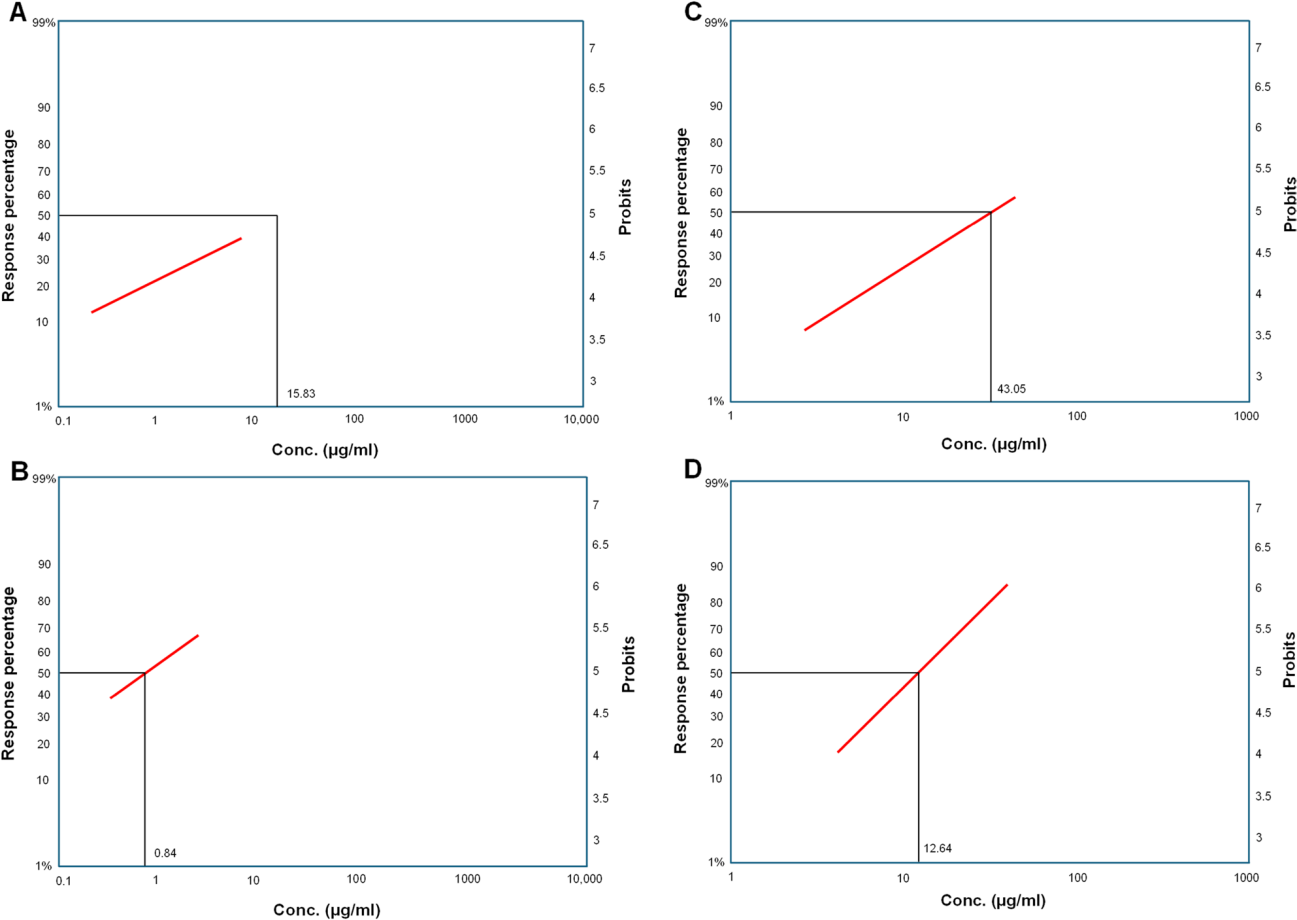
Dose-response regression plots of the two purified compounds on *Culex pipiens* larvae at two time intervals: (**A**) linear regression of deoxybrevanamide E at 24 h, (**B**) deoxybrevanamide E at 48 h, (**C**) physcion at 24 h, (**D**) physcion at 48 h

Regression analysis of deoxybrevianamide E and physcion revealed that the LC_50_ of these compounds at 24 h were 15.8331 and 43.056 µg/mL, respectively. The LC_50_ of these compounds at 48 h was 0.8421 and 12.6489 µg/mL, respectively. Figure 7, a multi-panel regression plot, demonstrated a concentration-related increase in larval mortality for both compounds. Pairwise statistical comparison shown in Table 7 revealed a significant increase in larval mortality between the 24-h and 48-h exposure periods for deoxybrevianamide E across all tested concentrations (*p* < 0.001). For physcion, mortality differences were concentration-dependent, with statistically significant temporal increases observed at concentrations ≥ 6.2 µg/mL. These findings demonstrate a clear time-dependent larvicidal effect. Overall, the bioassay results revealed that both compounds possessed larvicidal effects against third-instar larvae of *C. pipiens*. Notably, deoxybrevianamide E was more potent than physcion at equivalent doses.

**Table 7:** Pairwise statistical comparison of larval mortality between 24 h and 48 h exposure periods

Compound	Concentration (µg/mL)	Mortality 24 h (%)	Mortality 48 h (%)	*p*-value	Significance
Deoxybrevianamide E	0.315	12.86	39.92	0.00045	***
	0.625	17.50	46.92	0.00024	***
	1.25	23.14	54.08	0.00012	***
	2.5	29.67	61.12	0.00016	***
	5.0	36.94	67.81	0.00032	***
Physcion	3.25	7.84	15.30	0.303	ns
	6.2	14.41	29.55	0.049	*
	13	25.57	50.82	0.002	**
	26	39.11	70.64	0.00016	***
	52	54.12	85.66	0.00009	***

Statistical significance refers to pairwise comparison between 24 h and 48 h mortality (*p* < 0.05). *ns*not significant (*p* ≥ 0.05), *p < 0.05, **p < 0.01, ***p < 0.001

### Molecular docking analysis

The preliminary studies of the purified compounds (physcion and deoxybrevianamide E) were conducted to select the best insect protein receptors using a reverse docking approach. A variety of receptors were evaluated for each compound (See “Materials and Methods” section). The findings showed that all three substances had high binding affinities to neuron-related receptors, with values ranging from − 7.1 Kcal/mol to − 9.2 Kcal/mol. Physcion showed the highest docking score (− 8.9 kcal/mol) against the acetylcholinesterase (AChE). Glutamate-gated chloride channel (GluCl) was the ideal target for deoxybrevianamide E, with docking scores − 9.8 Kcal/mol (Table 8).

**Table 8:** The output binding energies (Kcal/mol) of compounds docked against several receptor proteins using the ReverseDock server

Receptors/ligands	Physcion	Deoxybrevanamide E
2bg9	−7.3 kcal/mol	−8.1 kcal/mol
6ary	−8.6 kcal/mol	−8.2 kcal/mol
3rhw	−7.1 kcal/mol	−9.2 kcal/mol
3ogn	−5.2 kcal/mol	−6.9 kcal/mol
5znt	−6.1 kcal/mol	−6.1 kcal/mol

**Fig. 8:**
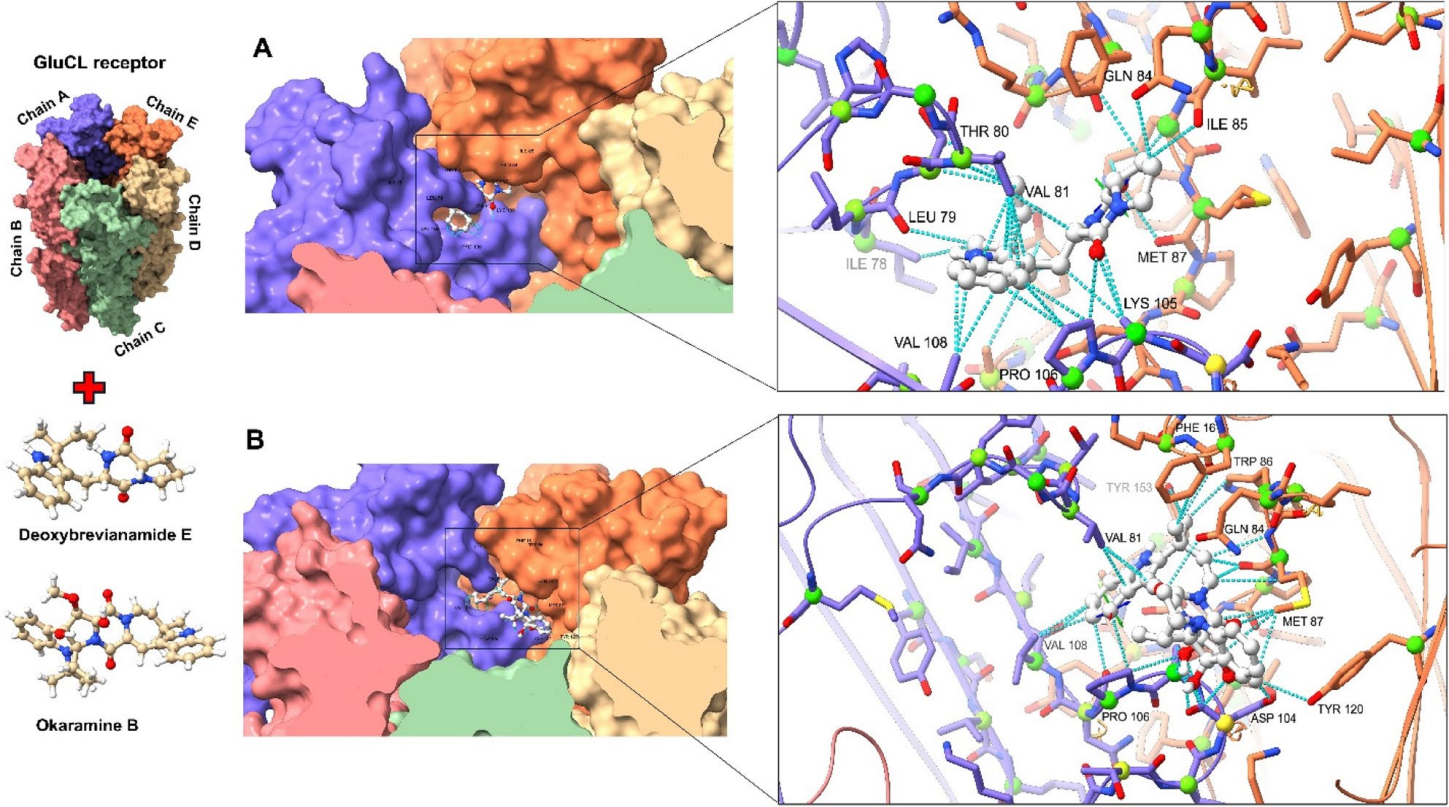
Molecular docking modeling between GluCl and deoxybrevanamide E (**A**) and Okaramine (**B**), showing the docked complex with binding contacts between ligands and the receptor

**Fig. 9:**
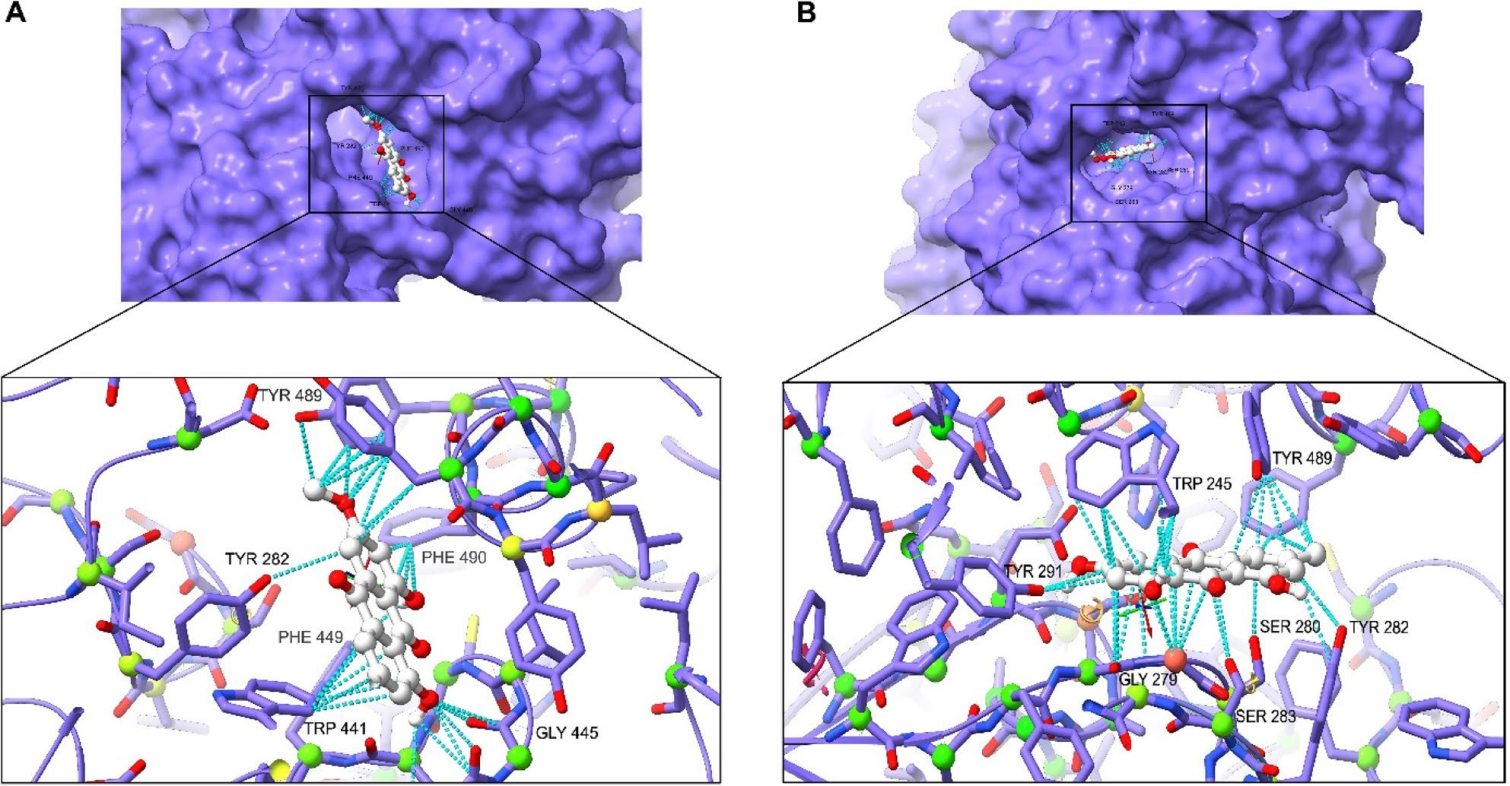
Molecular docking modeling between acetylcholinesterase (AchE) and physcion (**A**) and emodin (**B**) showing the docked complex with binding contacts between ligands and the receptor

Normal molecular docking was used to assess the compounds’ binding affinity and contacts with the highest anticipated receptors identified by inverse molecular docking, as well as to evaluate control compounds from the same chemical group as the tested ligands using CB-dock2. Deoxybrevianamide E was re-evaluated, along with two insecticidal indole alkaloid compounds (Okaramine A and Okaramine B), employed as controls against the GluCl receptor. The protein’s calculated free energy of binding to deoxybrevianamide E was − 9.5 Kcal/mol, while it scored − 9.1 and − 9.7 Kcal/mol for Okaramine B and A, respectively. At the interface of chains A and E, the docking analysis revealed that deoxybrevianamide E and Okaramine B share an active site and certain amino acid residues, including Val 81, Val 108, Gln 84, Pro 106, and Met 87 (Fig. 8). Okaramine A is located at the distinct active site at the interface of chains E and D (Supplementary Figure S6). Figure 9 displays the docked complex (ΔG = − 8.5 kcal/mol) between physcion and acetylcholinesterase (panel A), and the estimated free energy (ΔG = − 8.9 kcal/mol) between the emodin (control compound) and the acetylcholinesterase (panel B). The active sites of the two compounds are roughly the same, and they share common amino acids, including Tyr 489, Tyr 282, and Ser 280.

### Prediction of compounds’ toxicity

**Fig. 10:**
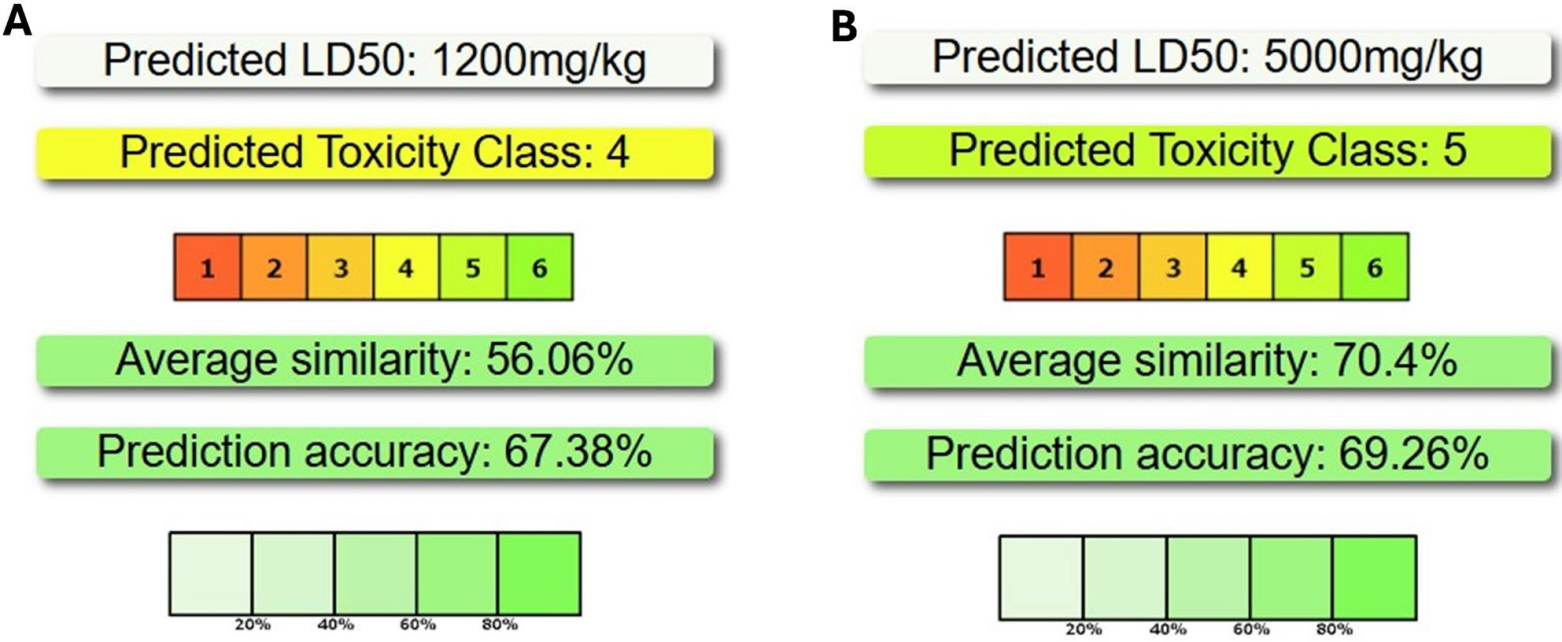
The predicted toxicity class, LD50, similarity, and accuracy of Deoxybrevianamide E (**A**) and Physcion (**B**)

The acute toxicity prediction results for oral administration within 24 h were analyzed using ProTox 3.0. Deoxybrevianamide E has a lower LD_50_ of 1200 mg/kg (class 4), a prediction accuracy of 67.3% **(**Fig. 10A**)**. Physcion exhibits a high LD50 value of 5000 mg/kg (class 5). Its predictions have a prediction accuracy of 70% **(**Fig. 10B**)**.

## Discussion

Fungal endophytes colonize plant tissues harmlessly and play a crucial role in promoting growth and protecting the host plant from harmful pests such as pathogenic microbes, nematodes, and insects [48, 49]. They produce a wide array of bioactive secondary metabolites that act as weapons to defend plants against biological threats, providing a sustainable alternative to synthetic pesticides [50]. These fungal-derived bioactive compounds minimize hazards to non-target species, reduce pesticide resistance, are safe for plants, and are also high-yielding [51–53].

The growing need for sustainable, low-toxicity insect vector control strategies has led to increased interest in endophytic fungal-derived products, particularly in light of the aforementioned advantages of these natural compounds as potential eco-friendly insecticidal agents [54–56]. In this context, this study screened crude extracts of six mangrove-associated endophytic fungal isolates for larvicidal effects against *C. pipiens*. It optimized the growth conditions to maximize bio-larvicidal metabolites yields by the most potent isolate (*Penicillium oxalicum* OQ231606.1). The study also isolated and purified bioactive compounds, elucidated their structures, and verified their efficacy via larval mortality bioassays. In silico molecular docking of the purified compounds with selected insect proteins was also performed to explore the potential targets within the larval body and clarify the potential larvicidal mechanism.

In the current study, preliminary screening of the crude extract of the six fungal endophytes revealed that all isolates exhibited larvicidal activity against third-instar larvae of *C. pipiens*. The observed mortality rates, ranging from 10 to 80%, showed a significant difference (*P* < 0.001), with *P. oxalicum* OQ231606.1being the most effective compared to the remaining isolates. Thus, it was selected for further optimization of larvicidal metabolites production. Consistent with this finding, the endophytic fungus *P. oxalicum* has been frequently reported as an effective biological control agent against various harmful pests, including plant pathogenic bacteria and fungi, insects, as well as drug-resistant human pathogens [57–60].

*C. pipiens* larvae exposed to the crude extract of *P. oxalicum* OQ231606.1 showed 80% mortality and significant morphological changes, including longer, pale larvae with ruptured integuments. The observed mortality and morphological deformations are likely due to disruption of insect metabolism caused by the fungal secondary metabolite, especially affecting nutrient absorption and hormonal regulation [61]. The current larvicidal activity of *P. oxalicum* aligns with previously reported effects of fungal secondary metabolites on mosquito physiology [58, 62]. The elongation and transparency of the larvae may result from the combined effects of fungal metabolites that may degrade or disrupt the synthesis of structural components. The loss of cuticle rigidity allows the body to stretch, while tissue lysis and hemolymph clarification increase transparency [63]. This pale color may also stem from a weakened melanization response, caused by early immune destabilization and rapid degeneration, leaving no time for darkening. The visible particles inside the larvae might be undigested metabolites from the fungal extract or immune complexes formed in response to it. These morphological changes align with histopathological results previously reported in other fungal-mosquito interactions, such as midgut necrosis in *Aedes aegypti* exposed to *P. citrinum* [64]. While the pesticidal effect of *P. oxalicum* is rarely recorded, its effectiveness in this study aligns with reports of *P. citrinum* and *P. chrysogenum* against *Ae. aegypti* [65]. Notably, the lack of previous studies on the effect of *P. oxalicum* against *C. pipiens* highlights the novelty of these findings.

Although optimized experimental design is crucial for improving fungal growth and metabolite production, traditional optimization methods are often labor-intensive, expensive, and time-consuming. Therefore, statistical strategies such as full factorial design (FFD) and central composite design (CCD) are efficient, reducing the experimental runs while revealing how variables affect responses. FFD tests all possible factor combinations, assessing main and interaction effects, aiding the design of predictive models to identify key parameters [66–68]. Subsequently, CCD further refines optimization with axial and center points, improving response surface accuracy with fewer experimental runs compared to FFD alone [69–71]. In the present study, a 2^4^ full factorial design, followed by a central composite design, was employed to optimize the production of larvicidal metabolites by *P. oxalicum* OQ231606.

The optimum growth and production conditions of *P. oxalicum* have been reported to vary significantly depending on the active compounds being produced, whether enzymes, antibacterial, antifungal, or other bioactive substances. The current finding observed an estimated optimum temperature of 28.66 °C for the production of the examined larvicidal compounds. Consistent with this finding, previously recorded *P. oxalicum* temperature range was from 25 to 36.5 °C. An optimum temperature of 36.5 °C was reported by Li et al. [72] for pectinase production optimized through RSM, 35 °C for cellulase production via OFAT methodology conducted by Li et al. [73], and 28 °C for cellulase in another study by Shah et al. [74]. Different optimum temperatures were also reported for fungal biomass production, with 25 °C and 27 °C being cited by Javaid et al. [75] and Kataria et al. [76], respectively, for biomass production. Additionally, 28 °C has been reported for the production of an antibacterial compound [77].

The optimum pH of the culture medium reported for *P. oxalicum* also varies depending on the target product, ranging from pH 5 for cellulase to pH 7.5 for xylanase production [73, 78]. The present results suggest that the optimal pH for production is about 6, aligning with the optimum pH recorded by Shah et al. [76] for cellulase production. Inoculum size and incubation periods also vary based on the desired product, as reported previously; *P. oxalicum*’s maximum cellulase activity was detected with an inoculum size of 10^6^ spores/mL and 5 days of incubation [76], while the optimum production of glucanase by *P. oxalicum* was recorded at the same inoculum size but with 10 days of incubation [79]. Our study found that *P. oxalicum* produces larvicidal compounds most effectively with ~ 1.2 × 10^6^ spores/mL (12% of the original spore suspension) and 20 days of incubation. This long incubation period is likely to allow full activation of biosynthetic gene clusters responsible for the production of these bioactive secondary metabolites, as reported by Mózsik et al. [80].

This study isolated and chemically characterized two major bioactive fungal metabolites, physcion and deoxybrevianamide E, from the crude ethyl acetate extract of the endophytic strain *P. oxalicum* OQ231606.1. The current NMR spectroscopic results of these two purified compounds align well with previously published NMR data for physcion [81] and deoxybrevianamide E [82], confirming the identity of both compounds. The results of larval mortality bioassay of both compounds revealed notable larvicidal effects of deoxybrevianamide E and physcion against third-instar larvae of *C. pipiens*, with LC_50_ of 15.8331 and 43.056 µg/mL, at 24 h, while the LC_50_ at 48 h was 0.8421 and 12.6489 µg/mL, respectively.

Both deoxybrevianamide E and physcion show time-dependent larvicidal activity, with deoxybrevianamide E being consistently significant across concentrations and physcion displaying a concentration-dependent response, aligning with established mosquito larvicidal bioassays and supporting the biological relevance of the LC₅₀ values [83, 84]. Overall, both compounds demonstrate potential as novel natural biopesticides, with deoxybrevianamide E, in particular, showing more potent activity and could serve as a lead compound for *C. pipiens* mosquito vector control strategies. The moderate activities of physcion also suggest possible synergistic or additive effects when used in combination.

Although conventional larvicides such as temephos (LC₅₀ ≈ 0.002–0.02 µg/mL) and *Bacillus thuringiensis* subsp. *israelensis* (Bti; LC₅₀ ≈ 0.01–0.1 µg/mL) generally exhibit higher absolute potency [85–87], the fungal-derived compounds identified in this study offer characteristic strategic advantages. Deoxybrevianamide E and physcion represent distinctive chemical scaffolds that may help overcome cross-resistance in mosquito populations resistant to temephos and other organophosphates, while their classification within indole alkaloid and anthraquinone families supports a favorable preliminary safety profile based on their extensive investigation and application in biomedical research. In addition, compared with Bti formulations that are susceptible to ultraviolet degradation, small-molecule fungal secondary metabolites may offer greater structural stability and predictable biodegradation, supporting their potential application in integrated vector management strategies [88–94].

In line with the present findings, the pesticidal property of physcion was previously reported against plant pathogenic fungi [95]. Physcion is also known to be produced by various fungal species, such as the marine-derived *Microsporum* sp [96] , as well as genetically modified strains of *Aspergillus terreus* and *A. oryzae* [95, 97]. Moreover, beyond the potential insecticidal effect of physcion reported in this study and its previously reported fungicidal activity, it also exhibits broad pharmacological profiles, including anticancer, antibacterial, anti-inflammatory, and antioxidant properties [95, 96, 98–101]. This wide spectrum of bioactivities underscores the great potential of physcion for use in biotechnological and agrochemical applications, including the production of bio-pesticides. Deoxybrevianamide E, categorized as a diketopiperazine indole alkaloid, is produced by various fungal species, mostly from the genera *Aspergillus* and *Penicillium*. Specifically, reported producers include *P. italicum*,* P. brevicompactum*, and *A. versicolor* [102, 103]. The biological activity of this compound has not yet been well explored. However, given its classification as an indole alkaloid and the broad range of bioactivities associated with this chemical group, its potential undiscovered biological activities could unlock novel applications in biotechnology and biological control.

In recent years, molecular docking has emerged as a crucial approach embedded in various scientific fields. This technique involves predicting the interactions between a chemical compound and a protein at the atomic level, enabling researchers to elucidate how these molecules behave at the active site of receptor protein residues [104–106]. In our study, the ReverseDock web server evaluates multiple insect receptors for their ability to interact with physcion and deoxybrevianamide E compounds. The results revealed the potential interaction between these compounds and protein receptors, with variations based on their docking scores. Several studies have explored the potential of natural compounds to inhibit multiple targets in insects [107, 108]. Despite these findings, AChE and GluCl were identified as potent receptors for these compounds. Acetylcholinesterase is essential for terminating the action of the neurotransmitter acetylcholine, which is a major potential target for insecticide development [107, 109]. Furthermore, GluCl is a key neurotransmitter receptor found exclusively in the nervous systems of invertebrates, making it a promising target for the discovery of selective insecticides [110, 111]. In comparison to other known insecticidal compounds, deoxybrevianamide E occupies the same active site as okaramine B. In 1989, okaramines, indole alkaloids, were identified from *Penicillium simplicissimum* and demonstrated to have insecticidal properties by targeting the GluGl receptor. Of these, okaramine B exhibited greater insecticidal efficacy than okaramine A [112–114]. On the other hand, the anthraquinone physcion shares the same active site as emodin. Emodin is an anthraquinone compound isolated from Aspergillus flavus that has high insecticidal activity against *Ae. aegypti*-AeA, *C. quinquefasciatus*-CuQ, and *Anopheles stephensi*-AnS larvae [115].

The bioactive compounds physcion and deoxybrevianamide E, produced by *P. oxalicum* OQ231606.1, exhibited potent larvicidal activity against *C. pipiens* while maintaining a favorable safety profile according to in *silico toxicity* predictions. Physcion was predicted to have a high LD₅₀ of 5000 mg/kg (GHS Toxicity Class 5, practically non-toxic), whereas deoxybrevianamide E was classified as Toxicity Class 4 (slightly toxic, LD₅₀ = 1200 mg/kg) [46, 47]. Importantly, the effective larvicidal concentration of deoxybrevianamide E (LC₅₀ = 0.84 µg/mL) is over a million-fold lower than its predicted vertebrate toxic dose, indicating strong selective toxicity.The ProTox 3.0 assessment further supports these findings, with prediction accuracies of 70% and 67.3% for physcion and deoxybrevianamide E, respectively, within the commonly reported performance range for state-of-the-art QSAR- and machine-learning-based toxicity models [46, 116–118]. This is reinforced by the established history of indole alkaloid and anthraquinone compounds in medical applications, which show no documented public health or environmental hazards [90–94]. Collectively, these results support the classification of these metabolites as potentially safe and selective bio-insecticides.

The eco-friendly potential of these compounds is suggested by their natural fungal origin, predicted low mammalian toxicity, and expected biodegradability, reducing the likelihood of environmental accumulation compared with persistent synthetic insecticides. Nevertheless, definitive confirmation of selectivity and ecological safety will require targeted non-target organism assays and environmental fate studies. These findings highlight the promise of endophytic fungal metabolites as sustainable larvicidal agents and justify further development for integrated vector management strategies.

It is noteworthy that the lack of prior research on the larvicidal effects of *P. oxalicum* and either of the two main bioactive compounds found in its crude extract (deoxybrevianamide E and physcion) emphasizes the novelty of these results. Ultimately, *P. oxalicum* OQ231606.1 can be a source of bioinsecticides, supporting sustainable insect management [119, 120]. However, thorough ecological risk assessments and long-term field studies are necessary to verify their safety across various ecosystems over time. Additionally, this research highlights the significant bioprospecting potential of endophytic fungi, especially those associated with the mangrove ecosystem, as sources of a wide range of biologically active natural products, including insecticidal agents.

## Conclusion

Given the paucity of works concerning the larvicidal activity of *Penicillium oxalicum*, this study is considered the first to report biolarvicidal activity of *P. oxalicum* and its metabolites, physcion and deoxybrevanamide E. The culture filtrate extract of *P. oxalicum* OQ231606.1 exhibited strong larvicidal activity against *Culex pipiens*, achieving up to 99.98% mortality under optimized conditions (pH 6, ~ 1.2 × 10⁶ spores/mL, 28 °C, 20 days). Physcion and deoxybrevanamide E were isolated, structurally characterized, and confirmed to possess potent larvicidal effects, with deoxybrevanamide E showing an LC₅₀ below 1 µg/mL at 48 h. Molecular docking studies suggested that physcion E likely binds to insect-specific targets, including acetylcholinesterase (AChE), with a binding affinity of -8.5 Kcal/mol. Meanwhile, deoxybrevanamide E could target glutamate-gated chloride channels (GluCl), with a binding affinity of -9.5 Kcal/mol, providing potential insights into their larvicidal mechanism of action. In addition, in silico toxicity predictions indicated low vertebrate toxicity, supporting their preliminary safety profile for further development.

These findings highlight *P. oxalicum* OQ231606.1 as a promising source of biopesticides and support physcion and deoxybrevanamide E as candidates for targeted vector control strategies. This work lays the groundwork for future studies, including large-scale metabolite production, experimental validation of target interactions, comprehensive toxicity and selectivity profiling, field efficacy trials, and evaluation of sustainable production substrates for potential commercial application.

## Supplementary Information

Supplementary Material 1.

## Data Availability

All data generated or analysed during this study are included in this published article and its supplementary information files.

## Abbreviations

AChE: Acetylcholinesterase
CCD: Central Composite Design
DMSO: Dimethylsulphoxide
EtOAc: Ethyl Acetate
FFD: Full Factorial Design
GluCl: Glutamate-gated Chloride channel
HPLC: High-Performance Liquid Chromatography
I.P: Incubation period
I.S: Inoculum size
LC_50_: Lethal Concentration 50%
MEA: Malt Extract Agar
MEB: Malt Extract broth
NMR: Nuclear Magnetic Resonance
OFAT: One-Factor-At-A-Time
PDA: Potato Dextrose Agar
PDB: Protein Database
RSM: Response Surface Methodology
Tem: Temperature
TLC: Thin-Layer Chromatography
UCSF: University of California San Francisco
VLC: Vacuum Liquid Chromatography
