# Supplementary material for: Exploring the efficacy of new potential bio-insecticides produced by Penicillium oxalicum against Culex pipiens larvae
Source: BMC Microbiol. 2026 Feb 25;26:297. doi: 10.1186/s12866-026-04783-5 (PMC13041276; doi:10.1186/s12866-026-04783-5)
Supplement: Supplementary file 1 — Supplementary Material 1. [file 12866_2026_4783_MOESM1_ESM.docx]

**Exploring the efficacy of new potential bio-insecticides produced by *Penicillium oxalicum* against *Culex pipiens* larvae**

**Hayam A. E. Sayed^1^ , Enas H. S. Ghallab ^2^, Ahmed M. Elissawy^3^, Peter F. Farag^1*^, Nevin A. Ibrahim^1^**

**^1^**  Department of Microbiology, Faculty of Science, Ain Shams University, Cairo 11566, Egypt.

**^2^** Department of Entomology, Faculty of Science, Ain Shams University, Cairo 11566, Egypt.

**^3^** Department of Pharmacognosy, Faculty of Pharmacy, Ain Shams University, Cairo 11566, Egypt.

***Corresponding author** **e-mail:** [peter_jireo@sci.asu.edu.eg](mailto:peter_jireo@sci.asu.edu.eg) (ORCID ID: 0000-0003-3329-7915)

**Tables**

**Table S1:** Coded Coefficients for the 2^2^ factorial design experiment

| **Term** | **Effect** | **Coef** | **SE Coef** | **T-Value** | **P-Value** | **VIF** |
| --- | --- | --- | --- | --- | --- | --- |
| Constant |  | 17.875 | 0.517 | 34.56 | 0.000 |  |
| pH | -4.375 | -2.188 | 0.517 | -4.23 | 0.001 | 1.00 |
| I.S | 0.375 | 0.187 | 0.517 | 0.36 | 0.722 | 1.00 |
| Tem | 14.500 | 7.250 | 0.517 | 14.02 | 0.000 | 1.00 |
| I.P | -13.500 | -6.750 | 0.517 | -13.05 | 0.000 | 1.00 |
| pH*I.S | 5.000 | 2.500 | 0.517 | 4.83 | 0.000 | 1.00 |
| pH*Tem | -2.875 | -1.437 | 0.517 | -2.78 | 0.013 | 1.00 |
| pH*I.P | 8.125 | 4.063 | 0.517 | 7.85 | 0.000 | 1.00 |
| I.S*Tem | -4.125 | -2.063 | 0.517 | -3.99 | 0.001 | 1.00 |
| I.S*I.P | 5.125 | 2.562 | 0.517 | 4.95 | 0.000 | 1.00 |
| Tem*I.P | -11.500 | -5.750 | 0.517 | -11.12 | 0.000 | 1.00 |
| pH*I.S*Tem | 1.250 | 0.625 | 0.517 | 1.21 | 0.245 | 1.00 |
| pH*I.S*I.P | -1.500 | -0.750 | 0.517 | -1.45 | 0.166 | 1.00 |
| pH*Tem*I.P | 2.875 | 1.437 | 0.517 | 2.78 | 0.013 | 1.00 |

**Table S2: 2^2^ factorial design Analysis of Variance of the assessed factors & their responses.**

| Source | DF | Adj SS | Adj MS | F-Value | P-Value |
| --- | --- | --- | --- | --- | --- |
| Model | 15 | 5972.50 | 398.17 | 46.50 | 0.000 |
| 1-Linear | 4 | 3294.25 | 823.56 | 96.18 | 0.000 |
| pH | 1 | 153.13 | 153.13 | 17.88 | 0.001 |
| I.S | 1 | 1.13 | 1.13 | 0.13 | 0.722 |
| Tem | 1 | 1682.00 | 1682.00 | 196.44 | 0.000 |
| I.P | 1 | 1458.00 | 1458.00 | 170.28 | 0.000 |
| 2-Way Interactions | 6 | 2198.50 | 366.42 | 42.79 | 0.000 |
| pH*I.S | 1 | 200.00 | 200.00 | 23.36 | 0.000 |
| pH*Tem | 1 | 66.12 | 66.12 | 7.72 | 0.013 |
| pH*I.P | 1 | 528.12 | 528.12 | 61.68 | 0.000 |
| I.S*Tem | 1 | 136.13 | 136.13 | 15.90 | 0.001 |
| I.S*I.P | 1 | 210.12 | 210.12 | 24.54 | 0.000 |
| Tem*I.P | 1 | 1058.00 | 1058.00 | 123.56 | 0.000 |
| 3-Way Interactions | 4 | 141.75 | 35.44 | 4.14 | 0.017 |
| pH*I.S*Tem | 1 | 12.50 | 12.50 | 1.46 | 0.245 |
| pH*I.S*I.P | 1 | 18.00 | 18.00 | 2.10 | 0.166 |
| pH*Tem*I.P | 1 | 66.12 | 66.12 | 7.72 | 0.013 |
| I.S*Tem*I.P | 1 | 45.12 | 45.12 | 5.27 | 0.036 |
| 4-Way Interactions | 1 | 338.00 | 338.00 | 39.47 | 0.000 |
| pH*I.S*Tem*I.P | 1 | 338.00 | 338.00 | 39.47 | 0.000 |

**Table S3: Coded Coefficients of the CCD experiment**

| **Term** | **Coef** | **SE Coef** | **T-Value** | **P-Value** | **VIF** |
| --- | --- | --- | --- | --- | --- |
| Constant | 97.67 | 2.55 | 38.23 | 0.000 |  |
| Blocks |  |  |  |  |  |
| 1 | -0.40 | 1.62 | -0.25 | 0.808 | 1.33 |
| 2 | -4.40 | 1.62 | -2.72 | 0.017 | 1.33 |
| pH | -2.83 | 1.28 | -2.22 | 0.045 | 1.00 |
| I.S | -1.00 | 1.28 | -0.78 | 0.448 | 1.00 |
| Tem | 6.58 | 1.28 | 5.15 | 0.000 | 1.00 |
| I.P | -8.92 | 1.28 | -6.98 | 0.000 | 1.00 |
| pH*pH | -25.21 | 1.19 | -21.10 | 0.000 | 1.05 |
| I.S*I.S | -25.71 | 1.19 | -21.52 | 0.000 | 1.05 |
| Tem*Tem | -13.71 | 1.19 | -11.47 | 0.000 | 1.05 |
| I.P*I.P | -14.96 | 1.19 | -12.52 | 0.000 | 1.05 |
| pH*I.S | 1.50 | 1.56 | 0.96 | 0.355 | 1.00 |
| pH*Tem | 0.12 | 1.56 | 0.08 | 0.938 | 1.00 |
| pH*I.P | 2.88 | 1.56 | 1.84 | 0.089 | 1.00 |
| I.S*Tem | -1.87 | 1.56 | -1.20 | 0.252 | 1.00 |
| I.S*I.P | 1.38 | 1.56 | 0.88 | 0.395 | 1.00 |
| Tem*I.P | -6.25 | 1.56 | -4.00 | 0.002 | 1.00 |

**Table S4: CCD design Analysis of Variance of the assessed factors and their responses.**

| **Source** | **DF** | **Adj SS** | **Adj MS** | **F-Value** | **P-Value** |
| --- | --- | --- | --- | --- | --- |
| **Model** | 16 | 38860.9 | 2428.8 | 62.02 | 0.000 |
| **Blocks** | 2 | 425.6 | 212.8 | 5.43 | 0.019 |
| **Linear** | 4 | 3165.0 | 791.2 | 20.21 | 0.000 |
| pH | 1 | 192.7 | 192.7 | 4.92 | 0.045 |
| I.S | 1 | 24.0 | 24.0 | 0.61 | 0.448 |
| Tem | 1 | 1040.2 | 1040.2 | 26.56 | 0.000 |
| I.P | 1 | 1908.2 | 1908.2 | 48.73 | 0.000 |
| **Square** | 4 | 34390.3 | 8597.6 | 219.56 | 0.000 |
| pH*pH | 1 | 17429.8 | 17429.8 | 445.10 | 0.000 |
| I.S*I.S | 1 | 18128.0 | 18128.0 | 462.93 | 0.000 |
| Tem*Tem | 1 | 5154.3 | 5154.3 | 131.63 | 0.000 |
| I.P*I.P | 1 | 6137.2 | 6137.2 | 156.73 | 0.000 |
| **2-Way Interaction** | 6 | 880.0 | 146.7 | 3.75 | 0.022 |
| pH*I.S | 1 | 36.0 | 36.0 | 0.92 | 0.355 |
| pH*Tem | 1 | 0.2 | 0.2 | 0.01 | 0.938 |
| pH*I.P | 1 | 132.3 | 132.3 | 3.38 | 0.089 |
| I.S*Tem | 1 | 56.2 | 56.2 | 1.44 | 0.252 |
| I.S*I.P | 1 | 30.3 | 30.3 | 0.77 | 0.395 |
| Tem*I.P | 1 | 625.0 | 625.0 | 15.96 | 0.002 |
| **Error** | 13 | 509.1 | 39.2 |  |  |
| **Lack-of-Fit** | 10 | 487.1 | 48.7 | 6.64 | 0.073 |
| **Pure Error** | 3 | 22.0 | 7.3 |  |  |
| **Total** | 29 | 39370.0 |  |  |  |

**Table S5:** Mortality percentages of 3^rd^ instar *Cx. pipiens* larvae exposed to different concentrations of deoxybrevianamide E after 24 and 48 hours.

| **Concentrations (μg/ml)** | **Average Mortality%**  **24 hrs** | **Average Mortality%**  **48 hrs** |
| --- | --- | --- |
| 0.315 | 12.8637 | 39.9207 |
| 0.625 | 17.4992 | 46.9155 |
| 1.25 | 23.1366 | 54.0849 |
| 2.5 | 29.6724 | 61.1229 |
| 5 | 36.9447 | 67.8147 |
| **Control** | 0.00 | 0.00 |
| LC_50_ (μg/ml) | 15.8331 | 0.8421 |
| **Slope ± SE** | 0.6660 ± 0.1499 | 0.5979 ± 0.1349 |

**Table S6:** Mortality percentages of 3^rd^ instar *Cx. pipiens* larvae exposed to different concentrations of physcion after 24 and 48 hours.

| **Concentrations (μg/ml)** | **Average Mortality%**  **24 hrs** | **Average Mortality%**  **48 hrs** |
| --- | --- | --- |
| 3.25 | 7.83932 | 15.2952 |
| 6.2 | 14.4079 | 29.5548 |
| 13 | 25.5746 | 50.8187 |
| 26 | 39.1080 | 70.6420 |
| 52 | 54.1209 | 85.6585 |
| **Control** | 0.00 | 0.00 |
| LC_50_ (μg/ml) | 43.056 | 12.6489 |
| **Slope ± SE** | 1.2623 ± 0.1561 | 1.7352 ± 0.1547 |

**Figures**

**Fig. S1. ^1^ H NMR spectrum of Compound 1 (Physcion)**

**Fig. S2. ^13^ C NMR spectrum of Compound 1**

**Fig. S3. COSY NMR spectrum of Compound 1**

**Fig. S4. ^1^H NMR spectrum of Compound 2 (Deoxybrevianamide E)**

**Fig. S5. ^13^C NMR spectrum of Compound 2**


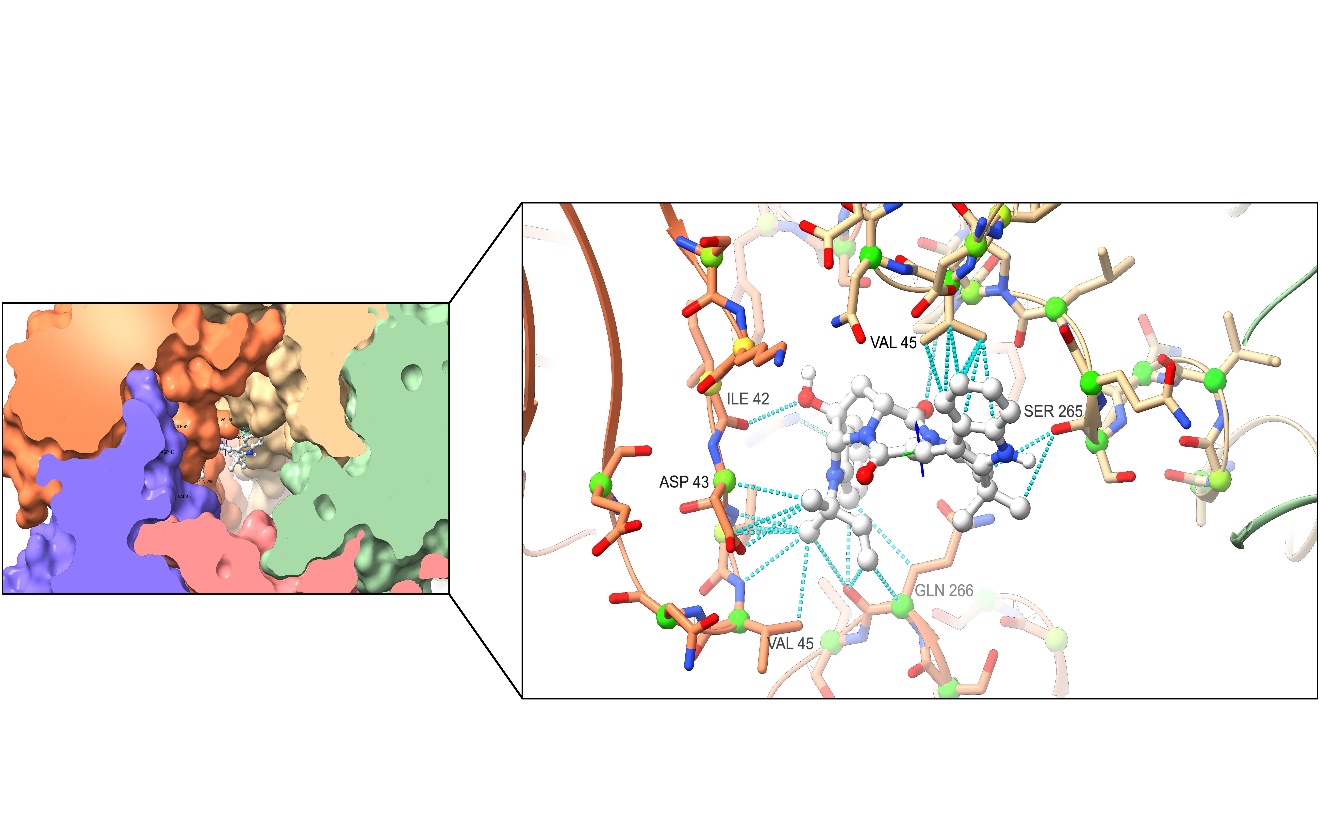


**Fig. S6:** Molecular docking modeling between GluCl and Okaramine A, showing the docked complex with binding contacts between ligands and the receptor.
